# String kernels for protein sequence comparisons: improved fold recognition

**DOI:** 10.1186/s12859-017-1560-9

**Published:** 2017-02-28

**Authors:** Saghi Nojoomi, Patrice Koehl

**Affiliations:** 1 Biotechnology program, University of California, Davis, 1, Shields Avenue, Davis, CA, 95616 USA; 2 Department of Computer Science and Genome Center, 1, Shields Avenue, Davis, CA, 95616 USA

**Keywords:** Protein sequence, Kernel, Alignment free methods

## Abstract

**Background:**

The amino acid sequence of a protein is the blueprint from which its structure and ultimately function can be derived. Therefore, sequence comparison methods remain essential for the determination of similarity between proteins. Traditional approaches for comparing two protein sequences begin with strings of letters (amino acids) that represent the sequences, before generating textual alignments between these strings and providing scores for each alignment. When the similitude between the two protein sequences to be compared is low however, the quality of the corresponding sequence alignment is usually poor, leading to poor performance for the recognition of similarity.

**Results:**

In this study, we develop an alignment free alternative to these methods that is based on the concept of string kernels. Starting from recently proposed kernels on the discrete space of protein sequences (Shen et al, *Found. Comput. Math.*, 2013,14:951-984), we introduce our own version, SeqKernel. Its implementation depends on two parameters, a coefficient that tunes the substitution matrix and the maximum length of *k-mers* that it includes. We provide an exhaustive analysis of the impacts of these two parameters on the performance of SeqKernel for fold recognition. We show that with the right choice of parameters, use of the SeqKernel similarity measure improves fold recognition compared to the use of traditional alignment-based methods. We illustrate the application of SeqKernel to inferring phylogeny on RNA polymerases and show that it performs as well as methods based on multiple sequence alignments.

**Conclusion:**

We have presented and characterized a new alignment free method based on a mathematical kernel for scoring the similarity of protein sequences. We discuss possible improvements of this method, as well as an extension of its applications to other modeling methods that rely on sequence comparison.

## Background

The overall principles behind the translation of a gene sequence into an active protein structure are theoretically well understood. The sequence of nucleotides that forms a gene is first translated into a sequence of amino acids, based on the genetic code. The corresponding linear chain of amino acids becomes active only after it folds into a three-dimensional shape, the so-called tertiary structure of the protein (though there is mounting evidence that there is a whole class of “unstructured” proteins that are biologically relevant [1]). However, while we can easily observe experimentally how the linear chain folds, we do not understand the principles underlying that transition. For instance, the rules that reveals the three-dimensional structure of a protein from its amino acid sequence have not yet been unraveled. Finding these rules is in fact one of the “holy grails” in molecular biology, namely the protein structure prediction problem [2, 3]. Efforts to solve this problem currently emphasize protein sequence analysis, as a consequence of the wealth of sequence data that is available. We know the primary sequences of many proteins, with currently more that 65,000,000 sequences in the TrEMBL database [4], we know in some cases the corresponding three-dimensional structures of these proteins, with more than 110,000 in the Protein Data Bank [5], and we have information on their biological activities, with more 550,000 sequences that have been annotated in SwissProt [6] (all numbers correspond to August 2016). However, we are still far from understanding all the information encoded in these databases. In this paper, we address the problem of protein sequence comparison in the context of protein fold recognition, and show that a new string kernel drastically improves the latter compared to traditional methods based on sequence alignment.

Amino acids are usually described using a one-letter code, and protein sequences are correspondingly represented as strings of letters. This representation has proved very useful, especially in the context of sequence alignment [7, 8] that is usually performed using string-matching algorithms [9]. Those methods represent the overwhelming majority of methods used for sequence comparisons in bioinformatics. When comparing two sequences, they proceed in two steps, first the generation of the alignment between the two sequences, then the derivation of a statistical score for that alignment. They rely on a weighting scheme to measure the cost of matching pairs of amino acids. Many such weights have been proposed, from substitution matrices derived from evolution models such as the PAM matrices [10] and the BLOSUM matrices [11], to matrices that capture physico-chemical properties of amino acids [12]. Using this score, an alignment is derived following a dynamic programming algorithm, either the local method of Smith and Waterman [13], or the global method of Needleman and Wunsch [14]. This alignment is then scored by summing the individual weights of the matching pairs of amino acids and adding penalties for the presence of gaps. It should be noted that this score is not a metric in sequence space. Statistical methods have been developed to assess the significance of such scores, both for gapped and non-gapped alignments (see for example [15]). Such statistical scores are widely used for the identification of homologous sequences or for fold recognition. It has been shown however that those scores are efficient for both tasks for sequences with high similarities, but often fail for dissimilar sequences (see for example [16]). Extensions to pair-wise alignment methods have been proposed to alleviate this problem, such as those based on multiple sequence alignments and profiles [17], and those based on Hidden Markov Models [8]. While those show improved sensitivity, they remain prone to the problems related to the construction and use of alignments.

To circumvent the shortcomings of the alignment-based methods described above, many “alignment-free” methods have been proposed over the past three decades (for review, see [18–20]). Most of these methods compute the frequencies of words of a fixed length, *k*, also denoted as *k-mers* or *k-grams*, depending on the authors. Once the frequency distribution functions of such *k-mers* have been computed for two sequences, the distance between those two sequences is assimilated to the distance between those distributions, using different definitions of distance [19, 21]. Other methods identify word matches of different lengths [22, 23]. One such method, the *average common substring approach*, identifies for each position *i* in one sequence the longest substring starting at *i* that is also present in the other sequence. The average lengths of those substrings over all positions *i* is a measure of similarity of the two sequences that can be converted into a distance [22]. All those methods are based on exact word matches. Exact matches however are bound to have limitations, due to strong correlations between amino acids at neighboring positions. A solution to the limitations of exact matches is the *spaced seeds* method that defines patterns with “*match*” and possible “*don’t care*” positions, using the vocabulary introduced by the authors [24–27].

Another class of alignment-free methods for comparing protein sequences that are directly relevant to this work includes string kernel based methods, originally defined in the context of support vector machines (SVM) [28]. SVM are machine learning algorithms that are designed to learn a rule for discrimination from a set of samples with two (or more) labels. This rule can then be applied to predict the label of any new sample. A key element of any SVM implementation is the kernel function that is used to quantify the similarity between any pair of samples. The simplest kernel function is the dot product between vectors of features that represent the samples. The first applications of SVM with a kernel function that is used to compute the similarity between two protein sequences were based on an extension of the dot product concept. Jakkola *et al* [29] used a generative Hidden Markov Model (HMM) on a set of proteins to generate a vector representation of each protein sequence (the so-called Fisher score vector). The kernel is then defined as a dot product between the corresponding Fisher vectors. Lodhi and colleagues introduced a string kernel that counts the number of occurrences of subsequences of a fixed length in the two strings that are compared [28]. The SVM-pairwise method [30] consists of describing each sequence with a vector of pairwise similarity scores for all domains in the training set (where the similarity score is the E-value of the Smith-Waterman pairwise sequence alignment), and defines the kernel to be the dot product between these vector representations. The spectrum kernel [31] and the mismatch kernel [32] measure the similarity between protein sequences by quantifying the number of similar short substrings (i.e. *k-mers* of fixed lengths, typically between 4 and 6 amino acids) they share. These two kernels bear similarity with the word-based alignment-free methods described above. The weighted degree kernels extend those kernels by considering weighted sums of the individual kernels obtained with fixed length *k-mers* [33 *,* 34]. The local alignment kernel of Saigo *et al* [35] was designed to mimic the score generated by a Smith and Waterman pairwise alignment method, with the proper mathematical foundations to guarantee that it is a true kernel. More recently, Smale and co-workers expanded the local alignment kernel by considering all possible alignments of *k-mers* between the two sequences of interest, for all possible *k* values, ignoring gaps when aligning the *k-mers* [36]. All the kernels listed here (as well as others that we have most likely missed), have been tested in classification problems as part of a machine learning algorithm (usually SVM), with various levels of success.

This paper draws from the concept of string kernels listed above. It describes an alignment-free method for protein sequence comparison that is based on the string kernel introduced by Smale and collaborators [36]. In contrast with the previous studies on string kernels, we do not include at this stage our string kernel into any learning algorithms. Instead, we assess directly its ability to classify proteins into structural folds based on sequence information only. We note that the string kernel we consider (which we refer to as SeqKernel) depends on two parameters, in addition to the substitution matrix it uses to score matches of pairs of amino acids (see below). We provide an exhaustive analysis of the effects of these two parameters on the performance of the kernel for fold recognition. Such an analysis, which is necessary as a first step to improving string kernel methods, was only partially included in the presentations of the equivalent kernels defined by Saigo *et al* [35], and by Smale and co-workers [36]. In the latter study for example, one parameter, *β* (see below for details), was fixed to 0.11, without further explanation, while the second parameter, *k*_*max*_, the maximum length of the *k-mers* considered, is set to a small number for computational considerations. Finding how those parameters influence the performance of the kernel is the main focus of this paper. To perform this analysis, we consider different datasets of proteins that belong to different structural folds, as defined by CATH [37]. These proteins were selected such that their sequences share little sequence similarities. We classified these proteins using the similarity measure provided by the string kernels. The classification is then compared with the corresponding results obtained using the scores of pairwise sequence alignments and the scores of structural alignments of those proteins. Ultimately, we have observed that sequence alone provides poor separation of the different folds. We show in contrast that with the right choice of parameters, use of the string kernel similarity measure significantly improves classification accuracy. In addition, we illustrate the use of SeqKernel to reconstruct a phylogenic tree of RNA polymerases, in line with the current development of alignment-free methods for phylogenomics that are designed to remove problems inherent to (multiple) sequence alignments (see for example [38 *–* 40]).

The paper is organized as follows. In “Methods” section, we describe the string kernel, while in “Results and Discussion” section we describe the databases we have generated to assess the performance of our string kernel for fold recognition. In the Results section, we provide a comprehensive analysis of the parameters that defined the kernel and finally we conclude the paper with a discussion on future developments of the string kernel approach.

## Methods

### A string kernel for alignment-free sequence comparison

The string kernel considered here is inspired by the convolution string kernels introduced by D. Haussler [41], adapted by Saigo *et al* [35] as the local alignment kernels, and later by Smale and co-workers [36]. We provide here the key elements of its construction. Readers should refer to the original papers for a more detailed presentation, notably for the proofs of the mathematical properties that are relevant to kernels in general.

**Notations** Let *X* be a finite set of size *n*. A kernel K is a symmetric function from *X*×*X* to $\mathbb {R}$ such that the Gram matrix *G* of size *n*×*n* defined by *G*(*i*,*j*)=*K*(*x*_*i*_,*x*_*j*_) is symmetric, positive, and definite. Let $\mathcal {A}$ be the set of the standard twenty amino acids found in proteins. A protein sequence *S* is a string of elements from $\mathcal {A}$. We note |*S*| the length of *S*.

**A kernel for amino acid pairs.** Let *SA* be a function from $\mathcal {A}\times \mathcal {A}$ to $\mathbb {R}$, such that *S* *A*(*i*,*j*) measures the similarity of the amino acids *i* and *j*, and let *SM* be the Gram matrix associated to *SA*. Examples of *SM* include the matrices representing the raw data of any BLOSUM matrices [42], namely the raw counts of how often amino acid *i* is substituted by amino acid *j* in a set of selected protein sequence alignments that is then normalized by considering its row sums *P*(*i*):

1 $$\begin{array}{@{}rcl@{}} P(i) = \sum_{j=1}^{20} SM(i,j)  \\ SM2(i,j) = \frac{SM(i,j)}{P(i)p(j)} \end{array} $$

We have checked that when *SM* is a raw count BLOSUM matrix, then *S* *M*2 is symmetric, positive, and definite. Note that when *SM* is such a raw count matrix, the corresponding BLOSUM matrix *BL* is defined as *B* *L*(*i*,*j*)=*r* *o* *u* *n* *d*(*l* *o* *g*_2_(*S* *M*2(*i*,*j*))), where *round* is the function that rounds a real number to its nearest integer. Given a strictly positive real number *β*, we define the function $K_{1}: \mathcal {A}\times \mathcal {A} \rightarrow \mathbb {R}$ as:

2 $$\begin{array}{@{}rcl@{}} K_{1}(i,j) = SM2(i,j)^{\beta}  \end{array} $$

*K*_1_ is a kernel function on $\mathcal {A}\times \mathcal {A}$, as long as *S* *M*2 is symmetric, positive, definite, and *β* is strictly positive. The same definition was used in [36].

**A kernel for comparing two strings of the same length.** Let *k* be a strictly positive integer and let $\mathcal {A}^{k}$ be the *k*-th Cartesian power of $\mathcal {A}$. An element of $\mathcal {A}^{k}$ is a string of *k* letters taken from $\mathcal {A}$, it is usually referred to as a *k-mer*, or a *k-gram*. Let *u* ^*k*^=(*u*_1_,…,*u*_*k*_) and *υ* ^*k*^=(*υ*_1_,…,*υ*_*k*_) be two *k-mers* in $\mathcal {A}^{k}$. The function $K_{2}^{k}$ defined by:

3 $$\begin{array}{@{}rcl@{}} K_{2}^{k}(u^{k},\upsilon{k}) = \prod_{i=1}^{k} K_{1}(u_{i},\upsilon_{i})  \end{array} $$

is a kernel on $\mathcal {A}^{k}$, the set of all *k-mers*. We note that $K_{2}^{k}$ is a convolution kernel [41].

**A kernel for computing protein sequence similarity.** Let *S*=(*s*_1_,…,*s*_*n*_) and *T*=(*t*_1_,…,*t*_*m*_) be two protein sequences; both are strings, with $S \in \mathcal {A}^{n}$ and $T \in \mathcal {A}^{m}$. Let *u* ^*k*^ and *υ* ^*k*^ be substrings of length *k* (i.e. *k-mers*) of *S* and *T* respectively. *u* ^*k*^ and *υ* ^*k*^ are considered contiguous, i.e. we do not allow gaps. There are therefore *n*−*k*+1 and *m*−*k*+1 such substrings in *S* and *T*, respectively. We define

4 $$\begin{array}{@{}rcl@{}} K_{3}^{k}(S,T) = \sum_{u^{k} \in S} \sum_{\upsilon^{k} \in T} K_{2}^{k}(u^{k},\upsilon^{k}) \end{array} $$

and

5 $$\begin{array}{@{}rcl@{}} K_{3}(S,T) = \sum_{k=1}^{p} K_{3}^{k}(S,T).  \end{array} $$

where the summation extends to *p*= min(*n*,*m*),with *n* and *m* the lengths of the two sequences that are compared. Finally, we define the correlation kernel $\hat {K}_{3}$ as:

6 $$\begin{array}{@{}rcl@{}} \hat{K}_{3}(S,T) = \frac{K_{3}(S,T)}{\sqrt{K_{3}(S,S)K_{3}(T,T)}}  \end{array} $$

Following [41] and [36], we make the following remarks:

i) The input kernel matrix *SM* is not a traditional substitution matrix, as it does not involve applying the logarithm function on the probability measures. While the latter is needed to make scores additive, a necessary condition for the use of dynamic programming algorithms in generating pairwise sequence alignment, it is not needed for the string kernel we use here. Note that this differs from the local alignment kernel, which is designed to mimic pairwise alignment.
ii) The kernel *K*_3_ is computed as an unweighted sum of the individual kernels $K_{3}^{k}$ that are computed with a fixed *k* value. We could have considered a weighted sum instead, similar in spirit to the weighted degree kernels [34]. We leave this option for future work.
iii) The summation in Eq. 5 extends to the length of the smallest sequence. This summation can be truncated however to only include *k-mers* up to a fixed length set to be *k*_*max*_. This will be discussed in details.
iv) $\hat {K}_{3}$ is a kernel as long as *S* *M*2 and *β* (which define *K*_1_) are definite positive, and strictly positive, respectively. Note that for any kernel *K*_1_ defined on $\mathcal {A}\times \mathcal {A}$, we can define a correlation string kernel $\hat {K}_{3}$.
v) As defined, $\hat {K}_{3}$ does not consider gap penalties, or even gaps. We consider this as an advantage, as it does reduce the number of parameters that defines $\hat {K}_{3}$.
vi) The string kernel $\hat {K}_{3}$ is a similarity measure in the space of sequences. Notice that for all sequences *S*, $\hat {K}_{3}(S) = 1$. This similarity measure can be transformed into a distance measure, using $D(S,T) = \sqrt {\hat {K}_{3}(S,S)+\hat {K}_{3}(T,T)-2\hat {K}_{3}(S,T)}=\sqrt {2-2\hat {K}_{3}(S,T)}$. *D*(*S*,*T*) takes values between 0 and $\sqrt {2}$.

### Implementing the string kernel

Equation 5 above leads to a simple, naive algorithm for computing the *K*_3_ kernel for two sequences *S* and *T*: for any length *k*, generate all *n*−*k*+1 and *m*−*k*+1*k-mers* in *S* and *T*, and perform the (*n*−*k*+1)(*m*−*k*+1) corresponding $K_{2}^{k}$ kernel evaluations using Eq. 3. Such an implementation however would come at a high computing cost, namely $O({nmk}_{max}^{2})$, where *n* and *m* are the lengths of *S* and *T* and *k*_*max*_ is the longest *k-mer* considered. We notice however that for any *k-mers* *u* ^*k*^ and *υ* ^*k*^, we have:

7 $$\begin{array}{@{}rcl@{}} K_{2}^{k}(u^{k},\upsilon^{k}) = K_{2}^{k-1}(u^{k-1}, \upsilon^{k-1}) K_{1}(u^{k},\upsilon^{k}) \end{array} $$

where *u* ^*k*−1^ is the string of length *k*−1 formed from the *k*−1 first components of *u* ^*k*^ (with a similar definition for *υ* ^*k*−1^). This simple recursion formula leads to a more efficient algorithm for computing the string kernel *K*_3_ of order *O*(*n* *m* *k*_*max*_). We note that this complexity remains large for protein sequence comparison.

### Datasets of protein sequences

Our first dataset includes 10,619 domains from the CATH [37] v4.0 domains, each with a CATH classification. As we focus on protein structure fold prediction, we consider the first three layers of the CATH classification, namely, Class (C), Architecture (A), and Topology (T). A set of structures with the same C, A, and T defines a fold. By using a set of structures with significant sequence diversity, we ensure that the data is duplicate-free. Such a filter when selecting the sequences also assures that the problem of detecting structural similarity is non-trivial. The 10619 structures were selected as follows: (i) Randomize the list of 235,858 CATH v4.0 domains; (ii) Start with the first domain on the randomized list, and remove from the list all domains that share significant sequence similarity with it (FASTA [43] SSEARCH E-value <10^−4^, with the alignments computed with the BLOSUM62 substitution matrix [42], and gap penalties of -11 (opening) and -1 (extension)). (iii) Repeat step (ii) with all domains in the list that have not yet been removed, until there are no domains left for selection. The set of 10619 domains resulting from this procedure is referred to as CATH40e4.

**Fig. 1:**
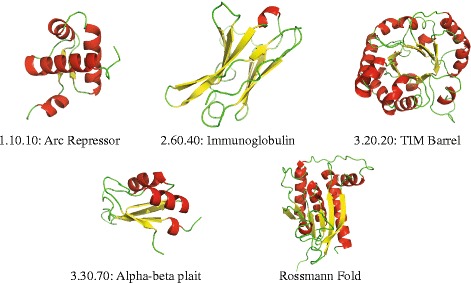
Representatives of the five fold classes in our test set CATH2833. The arc repressor mutant, subunit A fold (CATH 1.10.10) is a common orthogonal helix bundle, found, for example, in the Zb domain from the RNA editing human enzyme ADAR1 (CATH code 1xmkA00). The immunoglobulin-like fold is a *β* sandwich, found in many immunoglobulin-like proteins, such as the human MCG protein (CATH code 4unuA00). The TIM barrel is a very common *α*/*β* fold, shown in an isomerase of the parasite *Leishmania mexicana* (CATH code 2vxnA00). The *α*−*β* plait fold is a two-layer sandwich, shown here in a human nucleoprotein, HNRNP (CATH code 1l3kA01). The Rossmann fold is a very common three-layer sandwich fold in the mixed *α*−*β* class, found, for example, in the D-amino acic oxydase from *Rhodotorula toruloides* (CATH code 1c0pA01). All images were generated using Pymol http://www.pymol.org

There are 1363 folds in CATH40e4, many of which only include a single representative (734). To improve statistical analysis and remove possible biases from those folds with a low number of representative, we selected five of the most populated folds in CATH40e4 as a more specific test set, including at least one fold from each CATH class: CATH fold 1.10.10, a fully *α* fold (arc repressor, 381 members), CATH fold 2.60.40, a fully *β* fold (immunoglobulin-like, 555 members), and three alternating *α*/*β* folds: 3.20.20, (TIM-like, 251 members), 3.30.70, (two layer sandwich, 368 members) and 3.40.50 (Rossmann fold, 1278 members). The same five folds were previously used in other studies [44 *,* 45], but with different members as based on a different version of CATH. Overall, these five folds consist of a total of 2833 proteins (set CATH2833). Table 1 provides general information about the distributions of proteins in those five folds, while Fig. 1 illustrates the geometries of those folds, using the structure representatives defined by CATH.

**Table 1:** Statistics of the sequences included in the two datasets

		Dataset		
	CATH2833		CATH793	
CATH fold ID	N^a^	L (SD)^b^	N	L (SD)
1.10.10	381	79 (26)	36	135 (10)
2.60.40	555	110 (29)	130	140 (16)
3.20.20	251	294 (69)	2	157 (14)
3.30.70	368	182 (59)	52	141 (18)
3.40.50	1278	153 (77)	573	151 (17)

^a^Number of proteins in the fold

^b^Mean (standard deviation) of the lengths of the proteins in the fold

As seen in Table 1, the proteins in the five folds included in CATH2833 vary significantly in length. To assess the importance of length differences when computing the string kernel between two sequences, we generated a subset of CATH2833, CATH793, that contains all proteins whose lengths are between 120 and 180 amino acids. General information about those proteins is available in Table 1.

Finally, we extracted a second dataset from CATH40e4 that contains all folds with at least 40 representatives, excluding the five folds included in CATH2833. There are 40 of those folds, covering all three classes of proteins, and 13 architectures. This dataset contains a total of 3744 proteins and is referred to as CATH3744.

### ROC analysis of protein fold recognition

We quantify the effectiveness of a distance measure in identifying correctly if two sequences correspond to proteins that belong to the same CATH group using the receiver operating characteristic (ROC) analysis [46]. In the following, a “group” may be the class of the sequence (i.e. *α*, *β*, or *α*/*β*), the architecture of the corresponding protein structure, or the topology of the protein structure, as defined by CATH. A pair of protein sequences is defined as similar, or “positive”, if the sequences are members of the same group, and “negative” otherwise. All pairs of protein sequences in a dataset are then compared using a similarity measure. For varying thresholds of the measure considered, all pairs that fall below the threshold are assumed to be positive, and all above it are considered negative. The pairs that agree with the assumed standard are then called true positives (TP), while those that do not are deemed false positives (FP). A ROC analysis is set to measure the rate of TP as a function of the rate of FP. The “quality” of the similarity measure, namely its ability to separate positive pairs from negative pairs, is then scored using the Area Under the corresponding Curve, namely the AUC. An AUC score of 1 would indicate that all TP are detected first: this is consistent with an ideal measure. An AUC score of 0.5 indicates that TP and FP follow the first diagonal: they therefore appear at the same rate, indicating that the measure is not discerning.

When the number of groups considered is larger than two, we have performed two types of ROC analyses. In the first approach, we label a pair of proteins that belong to a given group as positive, independent of the group considered. In this case, an average behavior over all possible groups is derived. In the second approach, only pairs of proteins that belong to a specific group are called positive. This provides a group specific ROC analysis.

### Fold classification experiments

The ROC analysis described above ranks the distance measures of pairs of protein sequences and checks if this ranking is consistent with an existing classification; it is not designed to achieve the classification itself. We have expanded the ROC analysis to the actual issue of fold recognition by carrying out a second set of experiments. Each experiment involves a data set of protein sequences, *D*, a level of classification with groups, *G*, and a distance measure, *d*. We start by dividing randomly the sets of sequences in *D* into two subsets of approximately equal size. The first subset defines a training set, while the second subset corresponds to the test set. A test sequence is assigned a class by mapping it to the group of its nearest neighbor in the training set. Here the nearest neighbor is found by computing first the mean normalized distance between the test sequence and all sequences in the training set that belongs to a given group, for all groups in the training set, and then taking the smallest of those mean distances. The results are gathered in a confusion matrix, *C*, whose element *C*(*i*,*j*) corresponds to the number of test sequences from group *i* that have been classified as belonging to group *j*. The efficiency of the classifier *d* is then defined to be the quotient of the trace of the confusion matrix over the sum of all its elements. This quotient corresponds to the percentage of correctly classified sequences.

### Protein structure comparison

We have used STRUCTAL [47] to perform geometric alignments of two curves representing two protein structures. STRUCTAL starts with an initial alignment (a correspondence between residues of the two structures), and computes the rigid-body transformation that leads to the “best” geometric superimposition of the corresponding residues. It then identifies an optimal alignment for this superposition, using dynamic programming. The new alignment defines a new correspondence, which is used to superimpose the structures again. This procedure is then iterated until it converges to a local optimum that depends on the initial alignment. To alleviate biases due to that dependence, STRUCTAL repeats the iterative search using several different initial correspondences.

The traditional measure of similarity between two protein structures after optimal alignment is the root mean square displacement of atomic positions, also called cRMS for coordinate root mean square displacement, computed as:

8 $$\begin{array}{@{}rcl@{}} cRMS = \sqrt{\frac{\sum_{i=1}^{N} \text{dist}(a_{i},b_{i})}{N}} \end{array} $$

where N is the number of positions in the correspondence, and *a*_*i*_ and *b*_*i*_ are the Cartesian positions of two residues *a* and *b* from the two structures. The cRMS however is not a good measure of structural similarity [48]. Intuitively, a “good” measure of geometric, or structural similarity should favor alignments with many residues that are matched, low cRMS deviations, and few gaps. Unfortunately, these properties are not unrelated. For example, a lower cRMS deviation can always be achieved by selecting a smaller number of matches. In fact, given the fixed inter-CA distance there is the extreme case of alignments that only include two residues that have cRMS deviations of exactly zero. Also, the addition of gaps may lengthen the alignment without increasing the cRMS value. Different similarity measures have been proposed that attempt to balance these values in different ways. In this work, we have implemented the Structural Alignment Score, SAS:

9 $$\begin{array}{@{}rcl@{}} SAS = 100 \frac{cRMS}{N}, \end{array} $$

originally introduced by the authors of STRUCTAL [47].

### Reproducibility

The program SeqKernel described above, as well as all the datasets of sequences used in this study are available from the authors upon request.

## Results and Discussion

Two proteins with highly similar sequences almost always have similar structures. The reverse, however, is not always true. In a comprehensive analysis of the relationship between sequence similarity and structure similarity, Rost [16] has shown that pairs of proteins with similar structures possess, on average, only 8-10% sequence identity. This observation is at the root of the difficulties observed when attempting to classify proteins based on sequence information only. We have tested an alternative method to standard pairwise sequence comparison. We propose to use a string kernel that provides an alignment-free measure of the similarity of two protein sequences. We then use that measure to classify those protein sequences and compare the corresponding classification results with classifications derived from 3D structures and sequences only. Our aim is to parameterize the string kernel such that it performs better than sequence alignment based methods, mimicking to some extend the classifications derived from structure. We use CATH2833 and CATH793 as our main test sets. CATH2833 is a dataset of 2833 protein sequences that correspdond to the three main classes of CATH: one *α* fold, one *β* fold, and three *α*/*β* folds (see “Methods” section above). CATH2833 was designed in such a way that the sequences of any pair of proteins included have statistically no detectable similarity, by enforcing a FASTA [43] SSEARCH E-value >10^−4^. CATH793 is a subset of CATH2833 that contains those proteins whose lengths are limited to a small range, from 120 to 180 amino acids.

### Assessing the different protein sequence distances using receiver operator curve (ROC) analyses

**Fig. 2:**
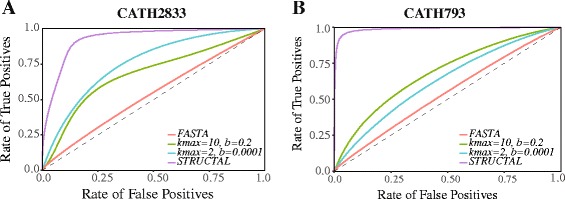
ROC analyses of three measures of protein similarity. We compare the efficiency of two sequence comparison methods, pairwise sequence alignment using SSEARCH from FASTA (*red*), the SeqKernel alignment free method introduced in this paper with two sets of parameters, (*k*_*max*_=2, *β*=0.0001) (*green*), (*k*_*max*_=10, *β*=0.2) (*cyan*), and the 3D structure alignment program STRUCTAL (*purple*) to detect fold similarity in two different sets of proteins, CATH2883 (panel **a**), and CATH793 (panel **b**). “True” relationships are defined according to CATH topologies. Curves close to the first diagonal (such as the ROC curve for FASTA) indicate poor performance, while the upper most curves (such as the 3D structure-based curve) indicate good performance

ROC analysis of protein fold recognition based on FASTA E-values for pairwise sequence comparison, SAS scores for 3D structure comparison using STRUCTAL (see “Methods” above), and two conditions for the alignment-free SeqKernel comparisons are shown in panels a and b of Fig. 2 for the datasets CATH2833 and CATH793, respectively. These ROC analyses are based on averaged behaviors over the five folds included in those two datasets (i.e. a pair of sequences is considered to be positive if they belong to the same fold, independent of this fold. Results broken down by fold are given in Tables 2 and 3 for CATH2833 and CATH793, respectively.

**Table 2:** Area Under the Curve (AUC) of ROC analyses for the FASTA, STRUCTAL, and SeqKernel distances for comparing protein sequences from CATH2833

	Distance				
CATH fold ID	N ^a^	FASTA ^b^	STRUCTAL ^c^	SeKernel(0.2,10) ^d^	SeqKernel(0.0001,2) ^e^
1.10.10	381	0.58	0.85	0.31	0.62
2.60.40	555	0.58	0.97	0.38	0.65
3.20.20	251	0.53	0.99	0.88	0.85
3.30.70	368	0.53	0.92	0.33	0.62
3.40.50	1278	0.53	0.92	0.77	0.77
All five folds	2833	0.54	0.93	0.69	0.76

^a^Number of proteins in the fold

^b^AUC based on FASTA E-value

^c^AUC based on STRUCTAL SAS score

^d and e^AUC based on SeqKernel distance, with (*β*,*k*_*max*_)=(0.2,10) (d) and (*β*,*k*_*max*_)=(0.0001,2) (e)

**Table 3:** Area Under the Curve (AUC) of ROC analyses for the FASTA, STRUCTAL, and SeqKernel distances for comparing protein sequences from CATH793

	Distance				
CATH fold ID	N ^a^	FASTA ^b^	STRUCTAL ^c^	SeKernel(0.2,10) ^d^	SeqKernel(0.0001,2) ^e^
1.10.10	36	0.75	0.91	0.61	0.61
2.60.40	130	0.55	0.98	0.44	0.54
3.30.70	52	0.51	0.89	0.45	0.47
3.40.50	573	0.57	0.98	0.69	0.62
All five folds	791	0.55	0.98	0.69	0.62

^a^Number of proteins in the fold

^b^AUC based on FASTA E-value

^c^AUC based on STRUCTAL SAS score

^d and e^AUC based on SeqKernel distance, with (*β*,*k*_*max*_)=(0.2,10) (d) and (*β*,*k*_*max*_)=(0.0001,2) (e)

The FASTA SSEARCH tool [43] is the implementation of a fast Smith and Waterman sequence comparison; it measures the similarity between two sequences either using directly the raw score of the alignment, or with a corresponding E-value. We have used the latter as a similarity measure. All SSEARCH alignments were performed using the BLOSUM62 substitution matrix [42], with gap penalties of -11 (opening) and -1 (extension). The ROC curves for this FASTA measure are marginally above the first diagonal, with AUC scores of 0.54 for both CATH2833 and CATH793. This behavior is expected, as by design all protein pairs in those datasets have little or no sequence similarity. The AUC values for the individual folds are very similar, ranging from 0.51 to 0.58, with one exception, fold 1.10.10 within the CATH793 dataset. We note however that this fold only contains 36 representatives in this dataset, making it the smallest set of all folds considered here. As such, this result is statistically unreliable.

Assignment of structural fold is expected to work best when it is based on 3D structural information. Indeed, the ROC curves obtained based on the SAS STRUCTAL scores illustrate excellent classification results, with AUC scores of 0.93 and 0.99 for CATH2833 and CATH793, respectively. We note that even with X-ray structure information the classification is not perfect, especially for CATH2833. This again is not a surprise. CATH is a semi-automatic classification of protein structures and some proteins are included in the same class based on more information than structure alone [37]. As such, two proteins may belong to the same class even though their structures are loosely similar. In addition, structural alignment programs work with heuristic algorithms and as such may miss the optimal alignment [48]. Finally, it is possible that a small fully *α* or fully *β* protein is found to be similar to an *α*/*β* protein, based on local alignment of the helical, or strand regions of the proteins. This is observed for example for proteins in fold 1.10.10 (the arc repressor all- *α* fold), with an AUC of 0.85 for CATH2833. That said, STRUCTAL scores based on X-ray structures still perform remarkably well.

The ROC curves based on the alignment free sequence comparisons obtained with SeqKernel are intermediate between the FASTA and STRUCTAL curves, for the two sets of parameters considered. Clearly, kernel similarity measures improve the classification of proteins into folds, especially for those proteins whose sequences bear little similarity. This improvement is significant, from an overall AUC score of 0.54 for CATH2833 with FASTA to AUC values between 0.63 and 0.76 for CATH2833 with SeqKernel, depending on the values given to the parameters *k*_*max*_ and *β*. Similar improvements are observed for CATH793. Interestingly, the improvements between FASTA and SeqKernel are not consistent over all types of folds considered. SeqKernel performs significantly better for proteins in the *α*−*β* class (folds 3.20.20 and 3.40.50) than for the full *α* fold (1.10.10) and for the full *β* fold (2.60.40). Of similar interest, there is a discrepancy between the results obtained on CATH2833 and CATH793: for the former, a significantly better overall AUC is observed when the pair (*k*_*max*_,*β*) is set to (2,0.0001), while for the latter, a better overall AUC is observed when the pair (*k*_*max*_,*β*) is set to (10,0.2). In addition, results using (*k*_*max*_,*β*)=(2,0.0001) are more consistent over the five folds considered. While differences are expected for different parameter values, the ranking of those parameter values is expected to remain the same on different datasets, especially here as CATH793 is a subset of CATH2833. This difference warrants a systematic study of the impact of the kernel parameters on its performance in fold recognition.

### Understanding the parameters *β* and *k*_*max*_ for the string kernel SeqKernel

**Fig. 3:**
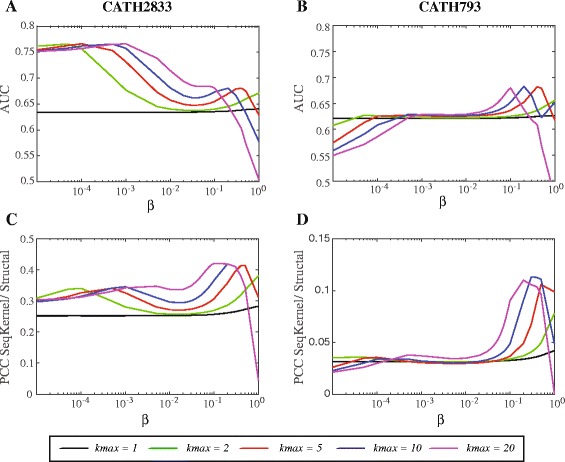
Parameterizing the string kernel SeqKernel. The string kernel defined in this paper is defined by two parameters, *β* and *k*_*max*_ (see text for details) We varied those two parameters in the respective ranges [10^−5^,1] and [1,20]; for each corresponding pairs of values, we applied the corresponding kernel to compute the similarities of all pairs of proteins in CATH2833 and CATH793 and checked the rankings of these similarities with the CATH classification of the proteins, using a ROC analysis. The corresponding AUC values are reported in panels **a** and **b**, respectively. High values of AUC indicate better fold recognition. Notice the different behaviors on the two datasets (panel **a** vs panel **b**). In panel **c** and **d**, we report in parallel the Pearson’s correlation coefficients between the kernel similarity measures and the STRUCTAL SAS values, for all pairs of parameters considered. As we assess the performance of SeqKernel in fold recognition, the SeqKernel values are expected to mimic the SAS scores, and therefore the larger the correlation coefficient, the better the performance of SeqKernel

The string kernel, SeqKernel, considered in this paper depends on two parameters, the coefficients *β* and *k*_*max*_. *β* is a power coefficient, i.e. it is used to compute the Hadamard power of the input substitution matrix *SM*. As it is used in a Hadamard power, all strictly positive values for *β* are possible, as the power matrix remains a Gram matrix. The parameter *k*_*max*_ defines the longest *k-mers* that are considered in the comparison of the two sequences. Note that the maximum value for *k*_*max*_ is the size of the smallest sequence. Intuitively, using large values for *k*_*max*_ are expected to help as the longer *k-mers* capture correlations in the protein sequences. However, as described in the implementation section, large *k*_*max*_ values come at a computational cost. We have tested a range of values for *β* from very small, 10^−5^, to relatively large, 1, and a range of values for *k*_*max*_, from 1 (i.e. single amino acid comparison) to 20. For pairs of values (*β*, *k*_*max*_) taken from their respective ranges, we computed the similarity scores for all pairs of proteins in CATH2833 and CATH793, and assessed the ability of those scores for fold recognition using ROC analysis. The resulting AUC scores are reported in Fig. 3. Note that the higher the AUC, the better the performance. In parallel, we also compared the string kernel similarity scores with the corresponding STRUCTAL SAS scores. As STRUCTAL was found to perform extremely well in the ROC analysis (see Fig. 2), the SeqKernel similarity scores should mimic the SAS scores for good fold recognition. We therefore computed the Pearson’s correlation coefficient PCC between the two sets of values, i.e. similarity scores and SAS values, for all pairs of proteins that belong to the same folds. The resulting PCC values are also reported in Fig. 3. Again, the higher the PCC, the better the performance.

The results on the CATH2833 dataset show two different behaviors depending on the *β* values: for very small *β* values (below 10^−3^), all the AUC=f(*k*_*max*_) curves reach the same plateau with a relatively high value of 0.76, while for larger values of *β* (>0.1), the same curves behave similarly but reach different maxima, with the *β* values corresponding to these maxima decreasing as *k*_*max*_ increases. Interestingly, the results on the CATH793 dataset are very different: while the same behavior is observed for the large values of *β*, SeqKernel provides poor fold recognition for very small *β* values. The key difference between the two datasets is that the latter includes proteins that cover a very small range of lengths. The discrepancy in behavior therefore hints to SeqKernel being able to pick differences in protein lengths for small *β* values. Indeed, as *β*→0, all the values in the kernel *K*_1_ tend to 1, and *K*_1_ becomes a matrix of ones. While this matrix does not correspond to a kernel (it is not positive definite), it can still be used in practice as input to SeqKernel. Using this matrix, the semi-kernel *K*_3_ can in fact be computed analytically:

10 $$\begin{array}{@{}rcl@{}} K_{3}(S,T) = \sum_{k=1}^{k_{max}} (n-k+1)(m-k+1)  \end{array} $$

Using this semi-kernel, we found that it performs with an AUC of 0.76 on the classification of the proteins in CATH2833. This high value of 0.76 therefore only reflects the differences in the distribution of lengths of the proteins in the five folds. Those differences are significant, see Table 1. While an interesting observation by itself, this is not the string kernel we are interested in. Indeed, Eq. 10 shows that it is independent of the actual sequences themselves, and only captures length differences; for example, the corresponding correlation semi-kernel takes the value of 1 for all pairs of sequences of the same lengths. These results suggest to use a kernel based on larger values of *β*. Figure 3 a and b indicate that any value of *k*_*max*_ is possible, pending that the proper value for *β* is chosen. We suggest using the pair (*β*,*k*_*max*_)=(0.2,10). Interestingly, the corresponding kernel leads to the same AUCs on the two datasets, CATH2833 and CATH793 (see above).

Comparisons of the kernel values with the STRUCTAL SAS values confirm this analysis, see panels c and d in Fig. 3. The PCC values between these two measures are found low for small values of *β*. In such conditions, the kernel was shown to mainly capture lengths, while the STRUCTAL scores are mostly independent of lengths. For larger values of *β*, the PCC values reach maxima for the same pairs of values (*β*,*k*_*max*_) than the AUC values.

### Choosing the input substitution matrix

**Fig. 4:**
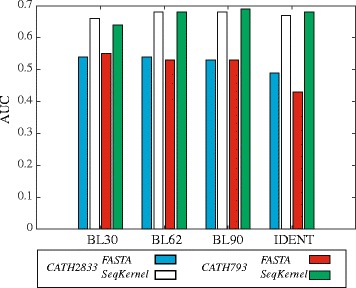
Choosing the substitution matrix. Comparison of the performances of FASTA and SeqKernel (*β*,*k*_*max*_)=(0.2,10), as measured by AUC values, for different input substitution matrices, namely the BLOSUM30 (BL30), BLOSUM62 (BL62), BLOSUM90 (BL90), and the Identity matrices. The two values for FASTA and for Seqkernel correspond to the two datasets CATH2833 and CATH793, respectively

There is one other parameter that defines the string kernel SeqKernel, namely the input substitution matrix. We compared fold recognition performance as measured by ROC analysis of both FASTA and SeqKernel (with *β* and *k*_*max*_ set to 0.2 and 10, respectively) on the two datasets CATH2833 and CATH793 for different input substitution matrices. Results are shown in Fig. 4. As a reminder, FASTA and SeqKernel use different matrices as input: FASTA uses a BLOSUM-like matrix, while SeqKernel uses its own count-based matrix. Those two matrices however are mathematically related by a simple *log* function. We note that there are little variations in the performance of both FASTA and SeqKernel as we change their input matrices. More surprisingly, SeqKernel performs quite well with the Identity matrix as input. This indicates that at least for the two datasets considered here, a strict score that does not favor replacement is good enough for fold recognition.

### Protein fold classification

We extended the ROC analyses of our sequence similarity measures to assessing their performances for actual fold recognition by performing a set of computational fold classification experiments. Note that we are not interested in establishing a classification for a family of protein structures: rather, we are interested in testing the ability of distance measures based on sequences to assign a protein to the structural class it belongs to. We perform this test using a series of computational experiments. For those experiments, we consider all folds included in Cath40e4 with at list 40 members, excluding the five folds considered in CATH2833, to eliminate possible biases as SeqKernel was parametrized based on these folds. There are 40 such folds, for a total of 3744 proteins, which we refer to as CATH3744. We consider three levels of classifications for those proteins, as defined by CATH: the Class level (3 such classes, *α*, *β*, and mixed *α*/*β*, the Architecture level (13 such architectures, 3 in the *α* class, 4 in the *β* class, and 6 in the mixed *α*/*β* class), and at the Topology (or fold) level (40 such topologies, 10 in the *α* class, 10 in the *β* class, and 20 in the mixed *α*/*β* class). Each experiment starts with those proteins, and a choice for the classification level. We pick at random half of the proteins of each group at the level considered, and define them as the *training set*. The remaining proteins form the *test set*. The training set is used to define the groups for the distance-based classifiers (see “Methods” for details). The experiment then proceeds by assigning a group to each protein in the test set. A protein is considered successfully classified if it is assigned to its actual CATH group (C, A, or T, based on the experiment considered). Experiments are repeated 10,000 times and the results averaged to remove potential bias in the choice of proteins that define the folds.

**Fig. 5:**
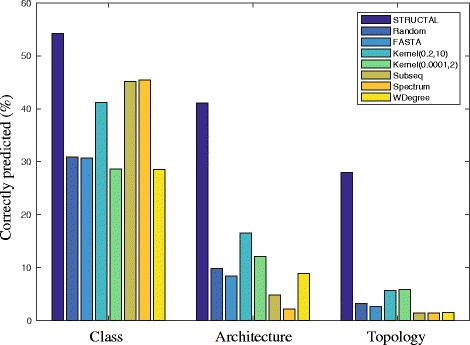
Classifying proteins in structural groups based on sequence-based distances. Three levels of structural classifications are considered, the C(lass), A(architecture), and T(opology) levels defined by CATH. Proteins are classified based on their shortest distance to a known group, where the distance is one of seven sequence-based distances between proteins, a RANDOM distance, a FASTA-based distance based on alignment, two distances based on the string kernel defined in this work, corresponding to two different parameter settings, (*β*,*k*_*max*_)=(0.2,10) and (*β*,*k*_*max*_)=(0.0001,2), and three other string kernel distances, Subseq [28], Spectrum [31], and WDegree, a weighted string kernel [33,34]). We also include results based on the STRUCTAL SAS scores; those results include structural information and should only be considered for reference. The classification accuracy (y-axis, in %) is computed as the ratio of proteins correctly classified over the total number of test proteins (see text for details)

For those fold classification experiments, we considered 8 possible distances between protein sequences. As a null reference, we assign a random value between 0 and 1 for the distance between two proteins. We call this distance RANDOM. On the other end of the spectrum, we include results based on the STRUCTAL SAS scores; those results directly reflect structural similarities. The third distance is based on the FASTA SSEARCH E-value. The fourth and fifth distances are computed with SeqKernel. As described in the previous section, there seem to be two regimes for SeqKernel, based on the values for the parameters *β* and *k*_*max*_. We picked one example from each regime, setting (*β*,*k*_*max*_) to (0.0001,2) and (0.2,10), respectively. The last three distances are based on other string kernels for comparing strings. We considered the subsequence string kernel introduced by Lodhi *et al* [28], Subseq, the Spectrum string kernel of Leslie *et al* [31], and WDegree, the weighted string kernel of Rätsch and colleagues [33,34]. For those last kernel distances, we used the implementations provided in the package Harry [49,50], with default parameters. Results of the classification experiments are given in Fig. 5.

Results from the reference RANDOM distances are on par with what is expected from the numbers of groups at each level of classification considered: 31% at the C level (1/3≈33*%*), 9% at the A level (1/13≈8*%*), and 3% at the T level (1/40≈3*%*). With no surprise, the results based on FASTA mirror those RANDOM results: as described above, there is no information in the FASTA distances by design. Also, without surprise, the classifications based on the STRUCTAL distances between structures are significantly more accurate than those based on sequence only. It is interesting however that even those results are far from perfect. At the C level for example, only 54% of the proteins are correctly classified. Most misclassification come from *α*+*β* proteins being classified as *α* or *β* proteins. This is not unexpected, as STRUCTAL performs a local structural alignment. The classifications obtained with SeqKernel with (*β*,*k*_*max*_) set to (0.2,10) are significantly more accurate than those observed with the other sequence-based distances, at least at the Architecture and Topology levels. This includes the results obtained with SeqKernel with (*β*,*k*_*max*_) set to (0.0001,2), confirming that a somewhat large value for *β* is preferred. Interestingly, the Subseq and Spectrum string kernels perform better at the Class level. It is likely that those two kernels capture the amino acid compositions of the sequences, which have been shown to be good discriminants of the protein structural class (as defined by CATH) [51,52].

### Alignment-free phylogeny reconstruction

A large number of biological modeling methods depend on accurate (multiple) sequence alignments. Protein structure prediction is one of those methods; it relies heavily on fold recognition. In the previous sections, we covered the use of alignment free methods for performing this task, comparing our string kernel SeqKernel with alignment-based methods. Another important modeling task that relies on sequence alignment is phylogenetic tree inference, a critical step in evolutionary studies. The majority of methods that perform this task follow a two step process, with the construction of a multiple sequence alignment followed by statistical tree inference [53,54]. These methods, though widely used, have known limitations related to uncertainties in the multiple sequence alignment [38,39,55–57]. Among all the approaches recently developed to alleviate those limitations, we note the alignment-free methods based on analyses of *k-mers* in the sequences that are compared [39,40,58,59]. As SeqKernel provides a mean to quantify the similarity between two sequences using such *k-mers*, we tested it on a simple toy problem of phylogenetic tree inference originally described by Thorne and Kishino [38]. To illustrate their methods for computing evolutionary distances between protein sequences, they considered 10 sequences of the second largest RNA polymerase subunit. We have repeated their analysis on the same sequences. These sequences include two eukaryotic pol I sequences, two eukaryotic pol II sequences, two eukaryotic pol III sequences, two archaebacterial sequences, a eubacterial sequence, and a chloroplast sequence; they are defined in Table 4.

**Table 4:** The ten RNA polymerases

Abbreviation	Protein	Species	Accession number	Length
SC1	RNA Pol I subunit RPA2	*Yeast*	RPA2_YEAST	1203
DR1	RNA Pol I subunit RPA2	*Drosophila megalonaster*	RPA2_DROME	1129
SC2	RNA Pol II subunit RPB2	*Yeast*	RPB2_YEAST	1203
DR2	RNA Pol II subunit RPB2	*Drosophila megalonaster*	RPB2_DROME	1129
SC3	RNA Pol III subunit RPC2	*Yeast*	RPC2_YEAST	1203
DR3	RNA Pol III subunit RPC2	*Drosophila megalonaster*	RPC2_DROME	1129
SUL	RNA Pol subunit *β*	*Sulfolobus acidocoldarius*	A0A0U3H235_9CREN	1126
MET	RNA Pol subunit B’	*Methano-bacterium*	RPOB1_METTW	1123
		*thermoautotrophicum*		
ESC	RNA Pol subunit *β*	*Escherichia coli*	RPOB_ECOSE	1342
SPI	Chloroplast RNA Pol subunit *β*	*Spinacia oleracea*	RPOB_SPIOL	1070

**Fig. 6:**
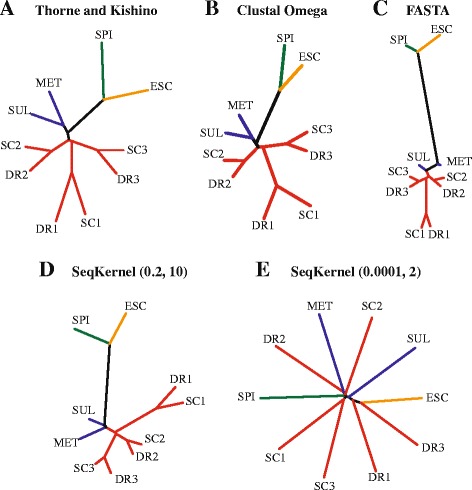
Inferred RNA polymerase phylogeny. Five different phylogenetic trees were constructed for a group of 10 RNA polymerases, using five different distance measures between their sequences: (**a**) the original distances proposed by Thorne and Kishino [38], (**b**) distances derived from a Multiple Sequence Alignment of the 10 sequences computed with Clustal Omega [60], (**c**) the BIT distances from FASTA SSEARCH tools, and the distances computed with SeqKernel with two different settings, namely (*β*,*k*_*max*_)=(0.2,10) (**d**) and (*β*,*k*_*max*_)=(0.0001,2) (**e**). Abbreviations for species names are provided in Table 4. Branches corresponding to eukaryotic sequences are colored red, those for archaebacterial sequences are shown in blue, and the eubacterial and chloroplast sequences are highlighted in orange and green, respectively

We considered 5 different distance matrices between those sequences. The first matrix is the original distance matrix proposed by Thorne and Kishino. The second matrix is derived from the multiple sequence alignment of those 10 sequences, computed with Clustal Omega [60], and converted into a distance matrix using the program ProtDist from the software package Phylip [61]. We used the Jones, Taylor and Thornton model of amino acid change [62] within ProtDist to compute the distances. The third matrix is based on the Bit scores of the sequence alignments generated by SSEARCH from FASTA, computed with Blosum62 as a substitution matrix and gap penalties of -11/-1 for opening/extension, respectively. The fourth and fifth matrices are derived from SeqKernel, with two settings for the parameters *β* and *k*_*max*_, namely (0.0001,2) and (0.2,10). We have built trees based on those five distance matrices using the programs Fitch and Drawtree from the Phylip package [61]. Fitch is an implementation of the Fitch and Margoliash [63] method for constructing trees from a distance matrix under the “additive tree model”. In this model, the distance between two sequences is expected to be equal to the sum of branch lengths between the sequences on the tree. These five trees are shown in Fig. 6.

With the exception of the tree generated from the distance matrix derived from the SeqKernel distances with *β* and *k*_*max*_ set to (0.0001,2) the trees are qualitatively similar to each other. As the 10 sequences have approximately the same lengths, and as SeqKernel with a small value of *β* basically captures differences in length, the exception is not surprising. This result however does reinforce our choice for (*β*,*k*_*max*_) to (0.2,10). To quantify the differences between the four remaining trees, we first scaled the distance matrices so that all distances ranged between 0 and 1, and then regenerated the trees. The resulting trees are then compared with the program TreeDist, implemented in the software package Phylip. TreeDist is based on the branch score distance of Kuhner and Felsenstein [64] to evaluate the similarity between two trees. We find that the distances between the original tree of Thorne and Kishino and the Clustal-based, FASTA-based, and SeqKernel-based trees are 0.3, 0.57, and 0.39, respectively. Note that the distance between the original tree and the SeqKernel-based tree when (*β*,*k*_*max*_) is set to (0.0001,2) is 0.99. While we cannot assess the meaning of the absolute values of these distances, and the significance of the differences between those values, we do notice that the original Thorne and Kishino tree resembles most the tree computed with the Clustal-based tree, and the tree computed with the method introduced here.

## Conclusions

The amino acid sequence of a protein encodes for its structure and ultimately its function in a cell. As such, sequence comparison remains one of the core tools used in many modeling methods in molecular biology that handle problems such as protein fold recognition and phylogenetic tree inference. We have shown that a string kernel that captures the similarity of all *k-mers* in two protein sequences provides an alignment-free method for fold recognition and phylogenetic tree reconstruction that performs well when its parameters have been selected appropriately. We refer to this string kernel as SeqKernel. It depends on two parameters, *β*, a power coefficient that modulates the values of the input substitution matrix, and *k*_*max*_, the maximum lengths of *k-mers* considered. We have performed a systematic analysis of the impact of those two parameters on the performance of SeqKernel in fold recognition experiments involving remote homologs. We have shown that on our test datasets, SeqKernel performs remarkably well for very small values of *β* (<10^−3^), independently of *k*_*max*_. With such small values of *β* however, SeqKernel is basically tuned to capture the difference in lengths of proteins, which is not of interest for fold recognition. We have shown that for larger values of *β*, there are pairs of values (*β*, *k*_*max*_) that provide significant performance in fold recognition. We suggest to use the pair (*β*,*k*_*max*_)=(0.2,10). Interestingly, in the first presentation of a kernel equivalent to SeqKernel [36], the authors advocate the use of *k*_*max*_=10, and referred to *β* as a mysterious parameter that they fixed to 0.11, i.e. close to the value of 0.2 that we propose here.

SeqKernel, just like any alignment-based sequence comparison method, depends on a score matrix, also called substitution matrix. Such a matrix provides a quantitative measure of the similarities of amino acids. We have shown that SeqKernel is not sensitive to the choice of this score matrix, at least on the two datasets CATH2833 and CATH793 used in this study. We note however that most of the score matrices, such as the BLOSUM matrices considered in this study, have not been optimized for the purpose of fold recognition. There has already been attempts to perform such optimization [65, 66]. In future studies, we will explore further the problem of defining an optimal score matrix within the context of SeqKernel.

This paper represents work in progress. It is dedicated to the understanding of the parameters that define the string kernel SeqKernel as well as to the illustration of its applications in fold recognition problems and phylogenetic tree inference. In the latter, we have only presented a small toy example to highlight that a alignment-free estimate of the distances between protein sequences can perform as well as a method based on multiple sequence alignment. Much work remains to be done before SeqKernel can become a commodity tool for sequence analysis. The scores computed with SeqKernel, while corresponding to a metric, do not include information on significance. The computing time for comparing two sequences using SeqKernel is high, suggesting that SeqKernel may not be used for large database searches. All the tests performed in this study were related to single domain proteins, or to specific domains within a protein. There is a need to expand the range of applications of SeqKernel to multi-domain proteins, as those are more the norm than single-domain proteins. Applications to phylogenomics need to be expanded and validated on a much larger scale. All those points will serve as research topics in our future studies.

## Abbreviations

AUC: Area under the curve
cRMS: Coordinate root mean square
ROC: Receiver operator curve
